# Classification of peripheral vitreoretinal interface lesions using spectral-domain optical coherence tomography with guidance of ultrawide field imaging

**DOI:** 10.3389/fnins.2025.1516919

**Published:** 2025-02-07

**Authors:** Weiwei Zheng, Ying Huang, Shanshan Qian, Bing Lin, Shenghai Huang

**Affiliations:** ^1^Eye Hospital and School of Ophthalmology and Optometry, Wenzhou Medical University, Wenzhou, Zhejiang, China; ^2^National Clinical Research Center for Ocular Diseases, Wenzhou, Zhejiang, China

**Keywords:** optical coherence tomography, ultrawide filed imaging, vitreoretinal interface, peripheral retinal lesions, peripheral vitreoretinal interface

## Abstract

**Purpose:**

This study aimed to classify peripheral vitreoretinal interface (VRI) lesions using optical coherence tomography (OCT) and to compare these findings with those obtained by ultra-widefield (UWF) pseudocolor imaging.

**Method:**

Peripheral OCT images of VRI lesions were obtained using spectral domain OCT system with a steerable probe guided by UWF images. Two independent investigators categorized the OCT images into four groups based on the extent of vitreoretinal traction and the presence of retinal breaks. Differences in OCT-based categorization between the same lesion types visualized by UWF imaging were also compared.

**Results:**

Of the total 82 patients, 105 peripheral lesions were included in this study. The inter-observer agreement for the classification of UWF and OCT images demonstrated good consistency, with kappa values of 0.949 ± 0.025 and 0.836 ± 0.042, respectively. In the OCT classification of VRI lesions, 18 (17.1%) cases were category A, 28 (26.7%) cases were category B1, 30 (28.6%) cases were category B2, and 29 (27.6%) cases were category C. Of the 37 vitreoretinal tuft lesions, 32.4% were classified as category B2 and 16.2% as category C, according to peripheral OCT classification. Similarly, 37.8% of 40 snail track and lattice degeneration lesions were classified as category B2, and 16.2% as category C.

**Conclusion:**

The VRI lesions can demonstrate considerable variability when visualized with peripheral OCT among the same lesion types visualized through UWF imaging. Classification of peripheral OCT images may provide a more effective evaluation of the risk of lesion progression.

## Introduction

The vitreoretinal interface (VRI) has been identified as the site of many peripheral retinal pathologies, such as snail track degeneration, lattice degeneration, retinal tufts, and peripheral retinal tears (Phillips et al., 2022; Shaimova, 2018; Sun et al., 2019; Cheung et al., 2022). The tractional forces which were caused by the dynamic interaction between the vitreous and the retina in the periphery play major role in the development of retinal tears and detachments (Phillips et al., 2022; Barak et al., 2012). Different types of peripheral retinal pathology indicate different potential risks for retinal tears or detachment, requiring different clinical decisions (Flaxel et al., 2020).

The indirect ophthalmoscope combined with scleral depression is gold standard for the examination of the peripheral retina (Natkunarajah et al., 2003). However, this examination requires qualified physicians with technical proficiency. The repeatability and comparability of the results are also limited. Ultrawide field (UWF) scanning laser ophthalmoscopy imaging, which can provide a field of view of over 200 degrees, helps improve the diagnosis and prognosis of peripheral retinal lesions (Quinn et al., 2019). Due to the transparency of the vitreous and the two-dimensional nature of UWF imaging, imaging of VRI lesions can be challenging, and it may not provide detailed information about the structure of the lesions. This makes it difficult to evaluate changes and assess the risk of progression during follow-up.

Optical coherence tomography (OCT) can provide high-resolution cross-sectional imaging of the vitreoretinal interface (VRI). It has become the gold standard for evaluating vitreoretinal disorders, such as epiretinal membrane, macular hole formation, and vitreomacular traction (Barak et al., 2012). OCT has a high axial resolution, which also allows for better evaluation of the peripheral retina. Peripheral OCT imaging has been used to image peripheral retinal lesions, such as snail-track degeneration, cystic retinal tuft, and retinal holes, with or without the guidance of UWF (Cheung et al., 2022; Li et al., 2022; Stanga et al., 2022). The structure of the VRI evaluated through cross-sectional retinal imaging of the peripheral retina using OCT, can provide valuable information for clinicians to develop follow-up plans and treatment strategies. However, OCT has a limited field of view, and relying solely on peripheral OCT is ineffective in accurately locating and imaging peripheral retinal lesions. To address this limitation, advancements in OCT technology have emerged. For instance, the Heidelberg Spectralis HRA-OCT incorporates a steering technique to achieve high-quality imaging of the peripheral retina (Choudhry et al., 2016). More recently, Silverstone (Optos) has provided an integrated UWF imaging and SS-OCT device, thereby improving the detection and characterization of peripheral retinal lesions (Pattathil and Choudhry, 2024; Koh et al., 2024; Kurobe et al., 2021).

While UWF imaging has been widely adopted in clinical practice for identifying peripheral retinal lesions (Kumar et al., 2021; Venkatesh et al., 2020; Fogliato et al., 2019), OCT has increasingly been utilized for imaging peripheral retinal lesions due to its ability to provide high-resolution cross-sectional views (Choudhry et al., 2016; Koh et al., 2024; Kurobe et al., 2021). To our knowledge, no standardized OCT-based classification system for VRI lesions has been established to date. This gap is particularly significant given the inherent limitations of UWF imaging, which lacks the depth resolution to fully characterize structural changes. In our observations, assessments of progression risks using peripheral OCT and UWF imaging have shown inconsistencies, highlighting the need for a more precise and standardized approach. Despite the growing clinical use of OCT for peripheral retinal imaging, research on its application for VRI classification remains notably limited. This study aims to address this gap by classifying peripheral VRI lesions using OCT and compare to the findings with UWF pseudocolor imaging.

## Methods

### Subjects

A single-center retrospective study was conducted. 95 eyes of 85 patients with peripheral retinal lesions were enrolled in this study from January 2021 to December 2022 at the Eye Hospital of Wenzhou Medical University. This study was approved by the Ethics Committee of the Eye Hospital of Wenzhou Medical University (2023-005-K-05-01). It adheres to the tenets of the Declaration of Helsinki.

All patients were subjected to slit lamp microscopy and mydriatic fundus examination. When there is suspicion of peripheral retinal lesions during mydriatic fundus examination, patients were required to undergo UWF and peripheral OCT examinations. Non-vitreoretinal interface lesions of the peripheral retina, such as microcystoid degeneration, paving stone degeneration, and peripheral drusen, were excluded (Cheung et al., 2022). Additionally, eyes with severe media opacities were excluded due to their potential to significantly degrade imaging quality.

### Imaging and analysis

Ultra-widefield (UWF) pseudocolor images were acquired using the Optos® California (Optos plc, Dunfermline, UK). Patients were asked to gaze at the desired direction in order to capture the UWF pseudocolor images of peripheral retinal lesions. Then the spectral domain OCT (SD-OCT) images were obtained by using the OCT SPECTRALIS® (Heidelberg Engineering GmbH, Heidelberg, Germany) under the guidance of UWF pseudocolor images (Choudhry et al., 2016). All SD-OCT images were performed by two experienced examiners (Z.W. and Q.S.). With the help of real-time scanning laser ophthalmoscopy (SLO) image and the guidance of UWF pseudocolor image, multiple B-Scan images which cross the lesion area were obtained by adjusting the patient’s gaze and direction of the OCT probe.

The UWF pseudocolor images were assessed to classify peripheral vitreoretinal interface abnormalities, drawing upon previous studies (Cheung et al., 2022). The classification criteria encompassed the following abnormalities: (1) vitreoretinal tuft, (2) snail track degeneration, (3) lattice degeneration, (4) retinal hole (operculated or atrophic), (5) retinal tear, (6) absence of any discernible lesions.

**Figure 1:**
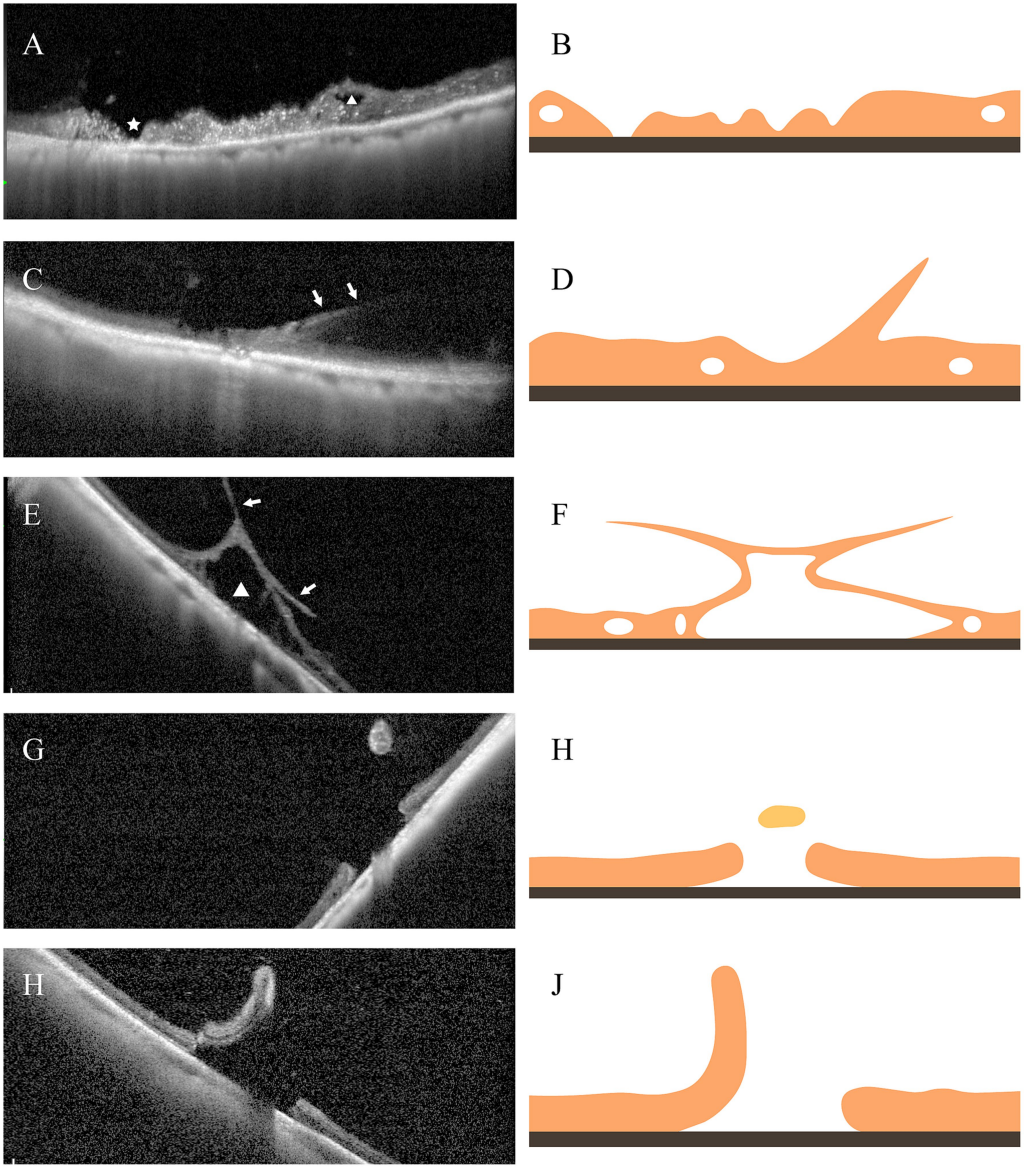
Illustration of different categories of the VRL lesions based on peripheral OCT imaging. **(A)** Category A: Peripheral retinal lesions lacking vitreoretinal traction, with intraretinal cavities (triangle) and retinal atrophy (asterisk). **(B)** Corresponding Schematic for **(A)**. **(C)** Category B1: Peripheral retinal lesions with evident vitreoretinal traction (arrow). **(D)** Corresponding Schematic for **(C)**. **(E)** Category B2: Peripheral retinal lesions exhibiting notable vitreoretinal traction (arrow) and significant intraretinal cystic spaces (triangle). **(F)** Corresponding Schematic for **(E)**. **(G)** Depicts an operculated macular hole, and **(I)** represents a retinal tear, both categorized as Category C. **(H,J)** represent the respective corresponding schematics.

Vitreoretinal traction and the presence of retinal breaks are critical imaging indicators in OCT assessments for predicting the risk of further retinal detachment (Barak et al., 2012; Choudhry et al., 2016; Konidaris et al., 2015). We propose categorizing peripheral OCT images into four primary groups based on the extent of vitreoretinal traction and the presence of retinal breaks. Figure 1 provides an illustration of the corresponding OCT images. Category A indicates the absence of apparent vitreous traction. Category B indicates apparent vitreous traction, without retinal break accompanied by an elevated edge. Further subcategories within Category B are distinguished based on the presence or absence of intraretinal cystic spaces induced by traction. B1 refers to the absence of apparent intraretinal cystic spaces, while B2 refers to the presence of apparent intraretinal cystic spaces. Category C represents cases of retinal breaks with an elevated edge.

### Interobserver agreement and statistical analysis

Two independent investigators (H.S. and Z.W.) classified UWF pseudocolor and peripheral OCT images separately. Any discrepancies in the data were meticulously addressed through reassessment and discussion with a senior researcher (L.B.). Inter-observer agreement between two readers was estimated by calculating the Cohen’s *kappa* coefficient using R software (version 4.2.1) with the vcd package (version 1.4.11). Descriptive statistics were used to summarize the data: continuous variables were reported as mean ± standard deviation (SD), while categorical variables were presented as frequencies and percentages.

## Results

### Baseline characteristics of eyes and patients

A total of 95 eyes from 85 patients underwent UWF and peripheral OCT imaging. However, three eyes (3.2%) from three patients were excluded from peripheral lesion imaging as OCT was unable to capture them. In all, 92 eyes from 82 patients were included in the study. Among these, both eyes of 10 patients were included and 10 eyes had more than 2 separate and independent lesions. Accordingly, a total of 105 peripheral lesions were acquired from the peripheral OCT images, which were then subjected to further analysis. Of the 82 study patients, there were 55 (67.1%) males and 27 (32.9%) females with a mean age of 27.8 ± 12.0 years (range, 14–69 years). The majority of patients (60/82, 73.2%) were 30 years old or younger. Of the 92 eyes, there are 43 (46.7%) right eyes and 49 (67.1%) left eyes. Of the 105 lesions, the distribution was: 34 (32.4%) in the superior quadrant, 36 (34.3%) in the temporal quadrant, 28 (26.7%) in the inferior quadrant, and 7 (6.7%) in the nasal quadrant. The patient’s Demographic Characteristics and Clinical Characteristics are summarized in Table 1.

**Table 1:** Demographic characteristics and clinical characteristics.

Characteristic	*N* (%) or Mean ± SD
Patients (*N* = 82)	
Age (years)	27.8 ± 12.0
Gender	
Female	27 (32.9%)
Male	55 (67.1%)
Eyes (*N* = 92)	
OD	43 (46.7%)
OS	49 (53.3%)
Quadrant of lesions (*N* = 105)	
Superior	34 (32.4%)
Temporal	36 (34.3%)
Inferior	28 (26.7%)
Nasal	7 (6.7%)

### Inter-observer agreement

The inter-observer agreement for the classification of UWF and peripheral OCT images was relatively good for both modalities, with kappa values of 0.949 ± 0.025 and 0.836 ± 0.042, respectively. The number of lesions with inconsistent classifications in UWF pseudocolor images (13/105, 12.4%) was higher compared to peripheral OCT images (4/105, 3.8%).

### OCT-based morphologic classification and ultra-widefield imaging classification

**Figure 2:**
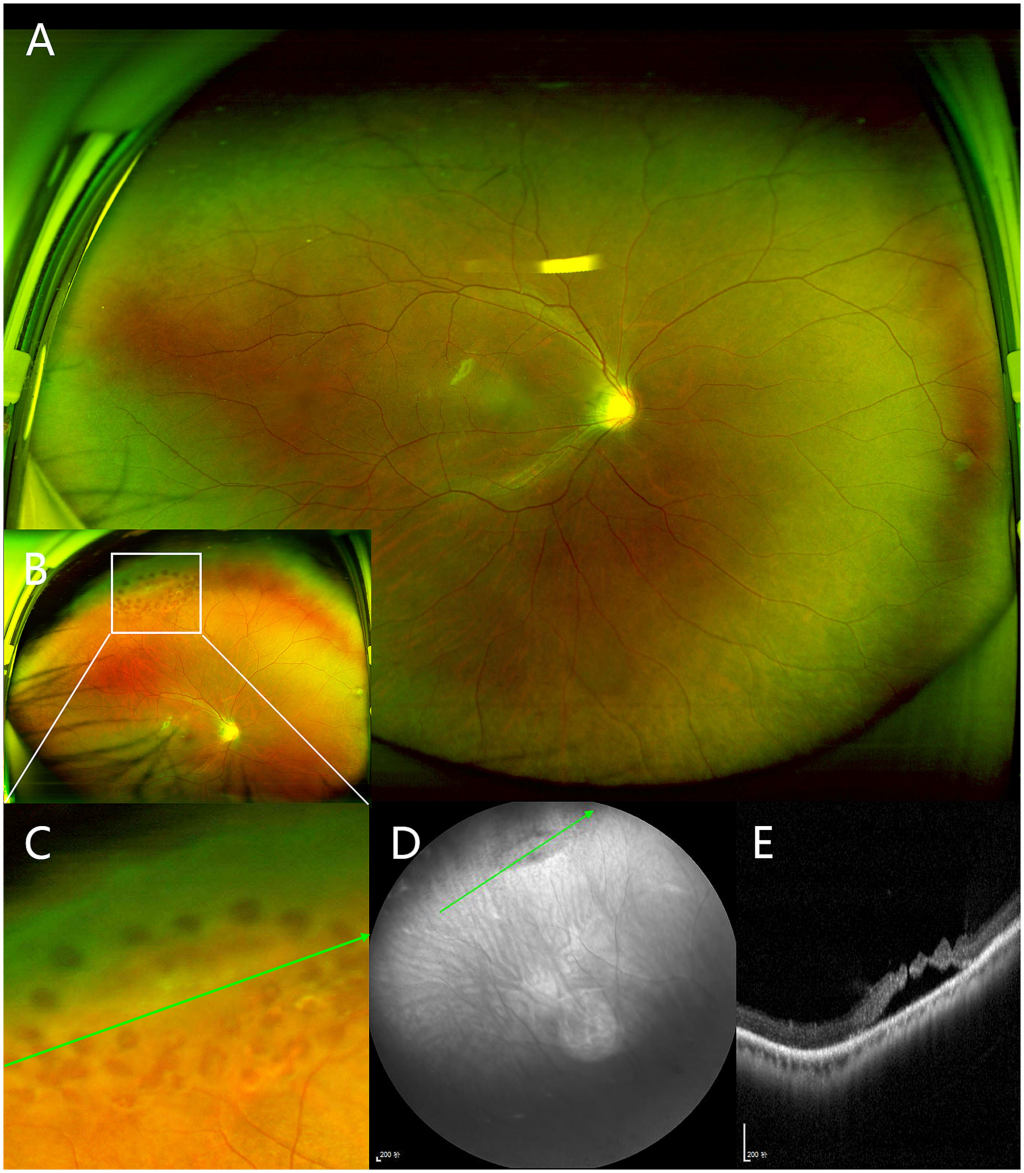
Peripheral vitreoretinal interface (VRI) lesion from a right eye of 18-year-old man. **(A)** The centered UWF image does not show any lesions in the superior retina. **(B)** The de-centered UWF image which was captured after peripheral laser treatment shows a snail track degeneration in the superior retina surrounded by peripheral laser spots, marked by the white box. **(C)** Magnified view of the indicated boxed area in **(B)**. **(D)** Near-infrared scanning laser ophthalmoscopy of lesion. **(E)** Peripheral OCT shows slit-like hole with peripheral retinal detachment, corresponding to the green arrows in **(C,D)**.

Among the VRL lesions, according to OCT classification, there were 18 (17.1%) cases of category A lesions, 28 (26.7%) cases of category B1 lesions, 30 (28.6%) cases of category B2 lesions, and 29 (27.6%) cases of category C lesions. Corresponding classification in UWF images were vitreoretinal tuft in 37 (35.2%) cases, snail track degeneration in 29 (27.6%) cases, lattice degeneration in 8 (7.6%) cases, retinal hole in 16 (15.2%) cases, and retinal tear in 1 (1.0%) case. However, there were 14 (13.3%) cases that could not be classified due to lack of imaging, with a considerable portion (9/14) missing a de-centered image in the lesion direction (Figure 2A). Table 2 shows a cross tabulation of OCT-based morphologic classification and UWF imaging classification for peripheral VRL lesions.

**Table 2:** Cross tabulation of OCT-based morphologic classification and ultra-widefield (UWF) imaging classification for peripheral vitreoretinal interface lesions.

Ultra-widefield imaging	Peripheral OCT				
	A	B1	B2	C	Total
Vitreoretinal tuft	8 (7.6%)	11 (10.5%)	12 (11.4%)	6 (5.7%)	37 (35.2%)
Snail track degeneration	3 (2.9%)	11 (10.5%)	10 (9.5%)	5 (4.8%)	29 (27.6%)
Lattice degeneration	1 (1.0%)	2 (1.9%)	4 (3.8%)	1 (1.0%)	8 (7.6%)
Retinal hole	2 (1.9%)	0 (0.0%)	1 (1.0%)	13 (12.4%)	16 (15.2%)
Retinal tear	0 (0.0%)	0 (0.0%)	0 (0.0%)	1 (1.0%)	1 (1.0%)
Absence of any discernible lesions	4 (3.8%)	4 (3.8%)	3 (2.9%)	3 (2.9%)	14 (13.3%)
Total	18 (17.1%)	28 (26.7%)	30 (28.6%)	29 (27.6%)	105

**Figure 3:**
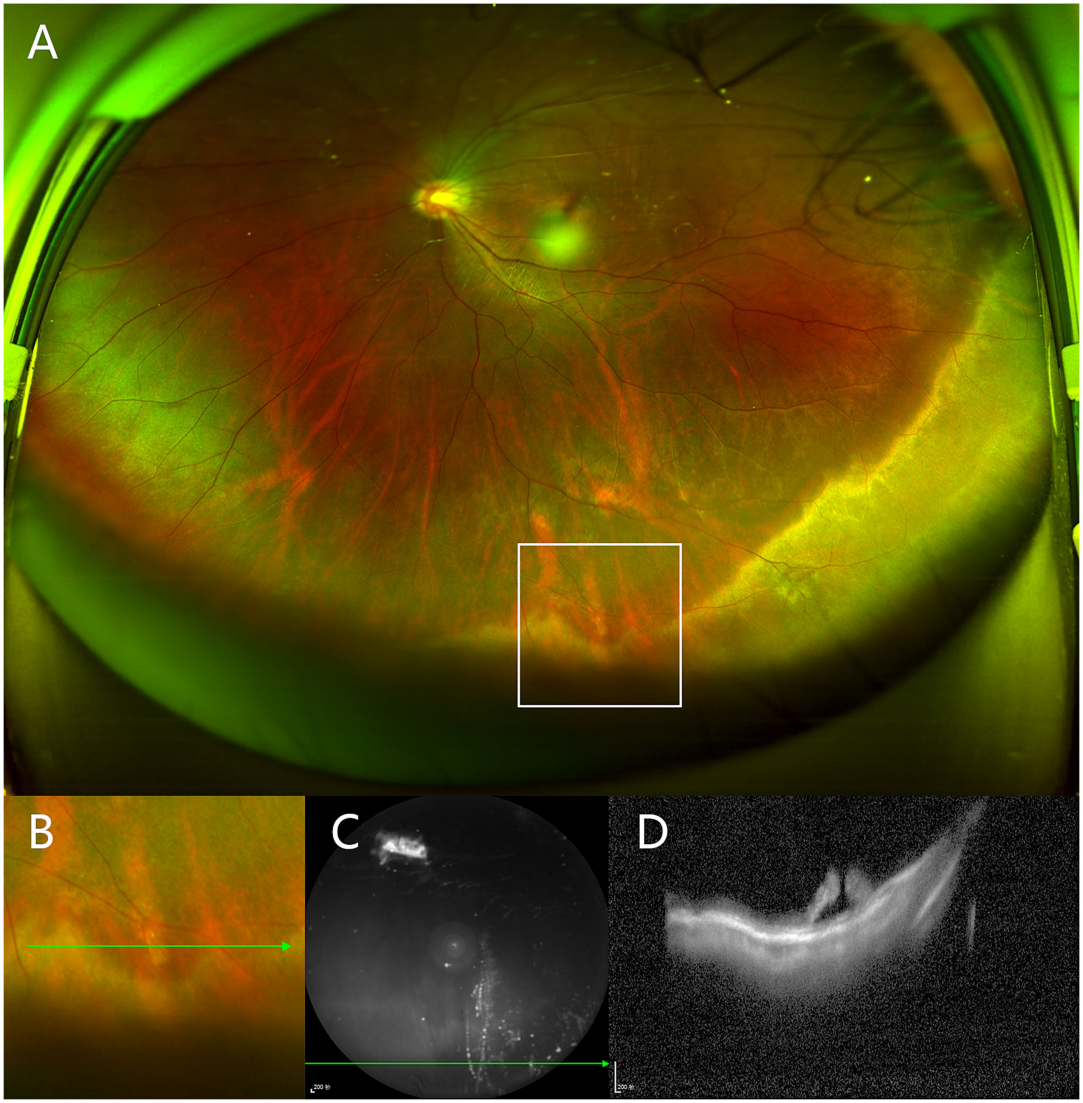
Peripheral vitreoretinal interface (VRI) lesion from a left eye of 19-year-old man. **(A)** UWF image shows a small vitreoretinal tuft lesion in the inferior retina, marked by the white box. **(B)** Magnified view of the indicated boxed area in **(A)**. **(C)** Near-infrared scanning laser ophthalmoscopy of lesion. **(D)** Peripheral OCT shows an edge-elevated retinal break, corresponding to the green arrows in **(B,C)**.

**Figure 4:**
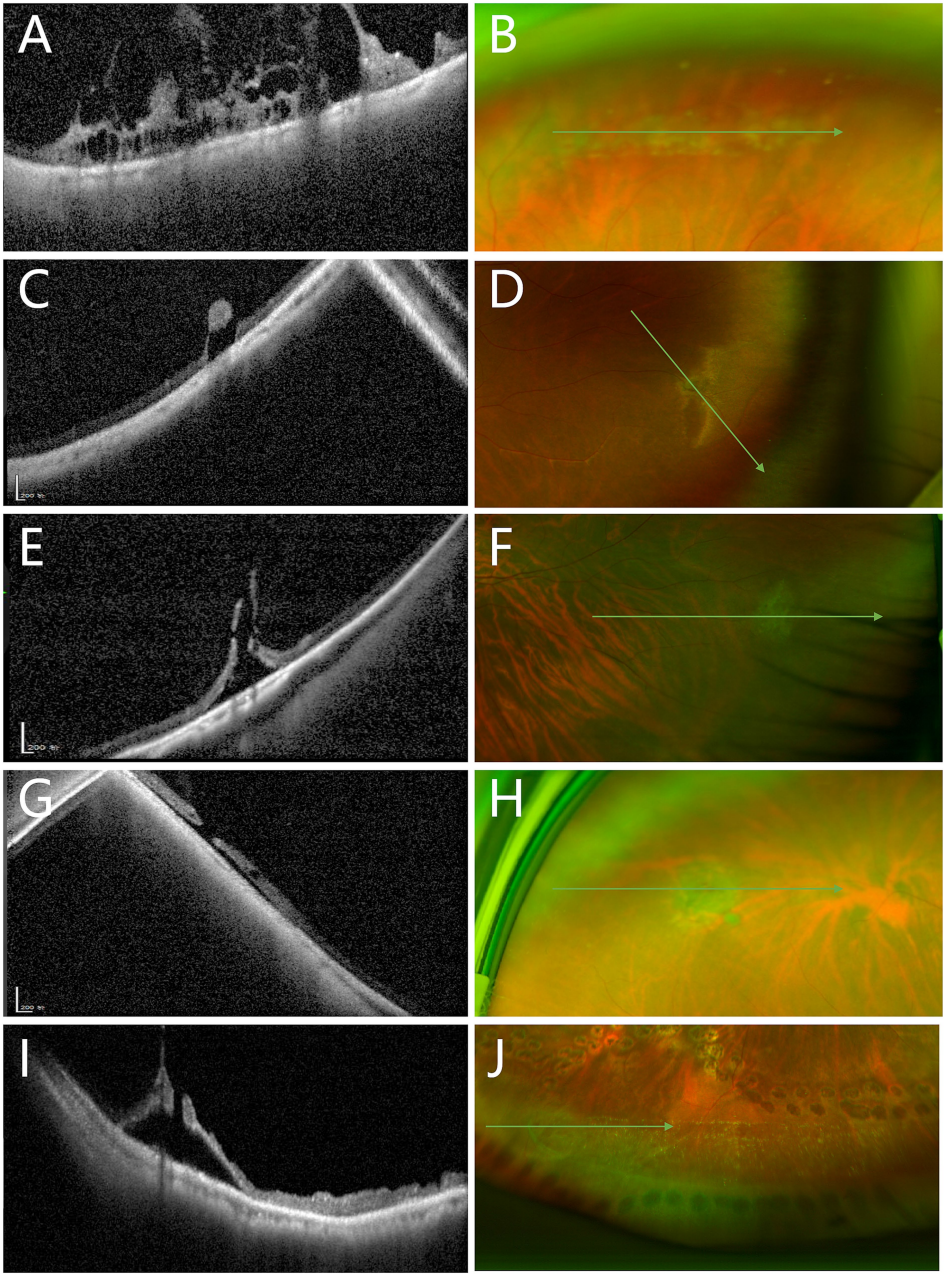
Comparison between peripheral OCT and UWF images in detecting vitreoretinal interface abnormalities. **(A)** OCT image shows marked vitreous traction with intraretinal cystic spaces (Category B2). **(B)** Corresponding UWF image reveals only snail track degeneration. **(C)** OCT shows retinal breaks with associated vitreous traction (Category C). **(D)** Corresponding UWF image shows only a vitreoretinal tuft. **(E)** OCT demonstrates elevated retinal breaks and vitreous traction (Category C). **(F)** Corresponding UWF image again shows only a tuft. **(G)** OCT reveals elevated retinal breaks (Category C). **(H)** Corresponding UWF image displays a retinal tear with an operculum. **(I)** OCT illustrates elevated retinal breaks with vitreous traction (Category C). **(J)** Corresponding UWF image shows snail track degeneration with a possible retinal break, lacking detailed traction information seen in the OCT image.

Among the 37 cases of vitreoretinal tuft lesions, 12 (32.4%) cases were classified as category B2 and 6 (16.2%) cases as category C based on peripheral OCT findings. The UWF image in Figure 3B showed a small vitreoretinal tuft lesion in the inferior retina. However, in the corresponding peripheral optical coherence tomography (OCT) image (Figure 3D), it presented as a retinal break with an elevated edge and was therefore classified as category C. Similarly, among all 37 cases of snail track and lattice degeneration lesions, 14 (37.8%) cases were classified as category B2 and 6 (16.2%) cases as category C based on peripheral OCT findings. The UWF image in Figure 2B showed a snail track degeneration in the superior retina, surrounded by peripheral laser spots. The corresponding peripheral OCT image (Figure 2E) revealed a slit-like hole with peripheral retinal detachment. Peripheral OCT often provides detailed insights into vitreoretinal interface conditions, including vitreous traction and retinal breaks, which may not be as clearly represented in UWF images (Figure 4).

## Discussion

Our study demonstrated the feasibility of imaging peripheral VRL lesions in a clinical setting using a steerable OCT probe guided by UWF pseudocolor image. The UWF pseudocolor imaging exhibits extensive utility in the detection of peripheral retinal lesions (Venkatesh et al., 2020; Mackenzie et al., 2007; Yang et al., 2020). The steerable OCT probe with guidance of UWF pseudocolor image has been successfully used to image the peripheral retina (Choudhry et al., 2016). Chu et al. (2019) successfully obtained OCT images in 91% of cases, while Sodhi et al. (2021) achieved successful imaging in 125 out of 134 consecutive cases. In our study, only 3 patients failed to achieve peripheral OCT imaging. Additionally, even in the 14 cases (out of 105) where lesions were not visualized under UWF pseudocolor image guidance, peripheral lesions could still be imaged with OCT using the vasculature as a reference in the UWF images. The low rate of imaging failure may be attributed to the relatively young mean age (27.8 years) of our patient cohort, as their clear refractive media and greater cooperativity facilitated peripheral retinal imaging. Therefore, a steerable OCT probe, in combination with UWF pseudocolor imaging, can effectively capture images of peripheral VRI lesions.

Peripheral OCT can better visualize the tractional forces of peripheral VRI lesions, as it allows direct cross-sectional imaging of the retinal structures involved. Classifying peripheral lesions with OCT can effectively assess the risk of progression toward vision-threatening retinal detachment. In our study, the proportions of various VRI lesions visualized on UWF pseudocolor images were similar to those reported in previous studies (Choudhry et al., 2016; Sodhi et al., 2021). However, we found the lesions appearing similar on UWF pseudocolor images can demonstrate considerable variability when visualized with peripheral OCT (Table 2). Although many VRI lesions are initially considered benign with minimal evidence of functional impact and a low possibility of progression over time (Flaxel et al., 2020; Byer, 1981; Byer, 1989), a subset of these lesions presents an elevated risk of retinal detachment. In our study, we identified lesions demonstrating evident vitreoretinal traction or potential for further progression (category B2 or C). Although only 1% of vitreoretinal tuft lesions may progress to vision-threatening retinal detachment (Byer, 1981), we found 32.4% of tuft cases demonstrated evident vitreoretinal traction with associated intraretinal cysts, thus categorized as B2. Additionally, 16.2% of tuft cases exhibited retinal breaks with elevated edges and were categorized as C. In Figure 3, a tuft lesion is shown on UWF pseudocolor imaging (Figure 3A), whereas OCT reveals a retinal break with an elevated edge (Figure 3D). Similarly, 37.8% of snail track or lattice degenerations were classified as Category B2, while 16.2% were classified as Category C. In Figure 2, a snail track degeneration is shown on UWF pseudocolor imaging (Figure 2B), whereas OCT slit-like hole with peripheral retinal detachment (Figure 2E), is also difficult to discern during indirect ophthalmoscopy. Therefore, some VRI lesions appearing benign on UWF pseudocolor imaging may still require further confirmation through peripheral OCT, facilitating a more precise risk assessment.

The classification of peripheral VRI lesions based on OCT allows for a more effective assessment of the risk of lesion progression. Abnormal vitreoretinal traction at the VRI lesion is a significant factor contributing to the progression of peripheral lesions (Phillips et al., 2022; Cheung et al., 2022). Therefore, in this study, OCT images lacking apparent vitreoretinal traction (Figure 1A) are classified as Category A, indicating a lower risk of progression. In accordance with the progression of traction lesions, Category B was further subdivided into B1 and B2, based on the presence of intraretinal cystic spaces induced by traction (Figures 1C,E). We believe Category B2, also known as acquired retinoschisis (Cheung et al., 2022), poses a greater risk of progression, especially in young myopic patients, necessitating more frequent follow-up. Finally, if the lesion presents a retinal beak with an elevated edge (Figures 1G,I), especially a retinal break with subretinal fluid, patients may benefit from retinal photocoagulation (Sodhi et al., 2021; Byer, 1989; Folk et al., 1989). We classified such OCT images as Category C. Thus, the classification of VRI lesions can improve the identification of high-risk lesions, enabling the adjustment of follow-up and treatment strategies in the clinic.

Classification of peripheral OCT images also demonstrated high inter-observer consistency. In previous studies, UWF pseudocolor imaging exhibited high specificity and sensitivity for detecting peripheral retinal lesions (Venkatesh et al., 2020; Mackenzie et al., 2007; Yang et al., 2020). Yang et al. (2020) showed that the kappa values for inter-observer consistency in detecting peripheral retinal degeneration and retinal holes using UWF pseudocolor images were 0.96 and 0.89, respectively. In our study, the overall kappa value was 0.836, which is similar to the study by Yang et al. For the classification of peripheral OCT images, the kappa value reached 0.949, revealing a level of agreement similar to that found for UWF pseudocolor images. The high degree of inter-observer consistency for the classification of OCT images makes it a reliable tool for clinical application.

Our findings highlight the critical role of OCT in identifying subtle structural changes in peripheral VRI lesions, such as vitreoretinal traction and retinal breaks, which are key indicators of lesion progression. The variability observed in OCT-based classifications, particularly in cases where UWF imaging failed to detect significant structural changes, underscores the potential of OCT to provide more precise prognostic information. For instance, the identification of intraretinal cystic spaces (Category B2) and retinal breaks with elevated edges (Category C) may serve as early markers for increased risk of retinal detachment. This suggests that OCT could be integrated into clinical practice as a prognostic tool, enabling earlier intervention and more tailored management strategies for patients with peripheral retinal pathologies. Future longitudinal studies are needed to validate these findings and establish standardized OCT-based criteria for predicting lesion progression and guiding clinical decision-making.

Our study has some limitations. Firstly, being an observational study, it restricts our ability to assess the risk of retinal detachment for different classifications of VRI lesions based on OCT. Secondly, the quality of some UWF pseudocolor images was compromised due to the lack of the de-centered image in the lesion direction (Figure 2A). This non-uniformity in imaging procedures results in some lesions being inadequately displayed or entirely omitted, introducing a potential source of bias into our findings. Additionally, due to the retrospective design, our study’s indirect dilated examination records were likely influenced by UWF pseudocolor and peripheral OCT findings rather than serving as an independent gold standard. Lastly, our study did not utilize wide-field OCT systems. Although wide-field OCT could facilitate easier acquisition of peripheral retinal lesions, the current steerable probe enables imaging of most lesions. Furthermore, our research aims to classify lesions based on OCT images. In future clinical applications, wide-field OCT systems will be adopted.

In conclusion, our study provided a classification of the VRI lesion based on peripheral OCT with guidance of UWF pseudocolor image. The VRI lesions, while appearing similar on UWF pseudocolor images, can demonstrate considerable variability when visualized with peripheral OCT. Our findings underscore the potential of peripheral OCT imaging, guided by UWF imaging, to provide a more detailed and standardized classification of peripheral VRI lesions. This approach could serve as a foundation for developing future clinical practice guidelines aimed at improving risk assessment and management strategies for patients with peripheral retinal pathologies.

## Data Availability

The raw data supporting the conclusions of this article will be made available by the authors, without undue reservation.
